# Clinical application of artificial intelligence in longitudinal image analysis of bone age among GHD patients

**DOI:** 10.3389/fped.2022.986500

**Published:** 2022-11-11

**Authors:** Lina Zhang, Jia Chen, Lele Hou, Yingying Xu, Zulin Liu, Siqi Huang, Hui Ou, Zhe Meng, Liyang Liang

**Affiliations:** ^1^ Department of Pediatrics, Sun Yat-Sen Memorial Hospital, Sun Yat-Sen University, Guangzhou, China; ^2^ Research Center for Healthcare Data Science, Zhejiang Lab, Hangzhou, China

**Keywords:** artificial intelligence, bone age assessment, growth hormone deficiency, children, China 05 bone age standard

## Abstract

**Objective:**

This study aims to explore the clinical value of artificial intelligence (AI)-assisted bone age assessment (BAA) among children with growth hormone deficiency (GHD).

**Methods:**

A total of 290 bone age (BA) radiographs were collected from 52 children who participated in the study at Sun Yat-sen Memorial Hospital between January 2016 and August 2017. Senior pediatric endocrinologists independently evaluated BA according to the China 05 (CH05) method, and their consistent results were regarded as the gold standard (GS). Meanwhile, two junior pediatric endocrinologists were asked to assessed BA both with and without assistance from the AI-based BA evaluation system. Six months later, around 20% of the images assessed by the junior pediatric endocrinologists were randomly selected to be re-evaluated with the same procedure half a year later. Root mean square error (RMSE), mean absolute error (MAE), accuracy, and Bland-Altman plots were used to compare differences in BA. The intra-class correlation coefficient (ICC) and one-way repeated ANOVA were used to assess inter- and intra-observer variabilities in BAA. A boxplot of BA evaluated by different raters during the course of treatment and a mixed linear model were used to illustrate inter-rater effect over time.

**Results:**

A total of 52 children with GHD were included, with mean chronological age and BA by GS of 6.64 ± 2.49 and 5.85 ± 2.30 years at baseline, respectively. After incorporating AI assistance, the performance of the junior pediatric endocrinologists improved (*P* < 0.001), with MAE and RMSE both decreased by more than 1.65 years (Rater 1: ΔMAE = 1.780, ΔRMSE = 1.655; Rater 2: ΔMAE = 1.794, ΔRMSE = 1.719), and accuracy increasing from approximately 10% to over 91%. The ICC also increased from 0.951 to 0.990. During GHD treatment (at baseline, 6-, 12-, 18-, and 24-months), the difference decreased sharply when AI was applied. Furthermore, a significant inter-rater effect (*P* = 0.002) also vanished upon AI involvement.

**Conclusion:**

AI-assisted interpretation of BA can improve accuracy and decrease variability in results among junior pediatric endocrinologists in longitudinal cohort studies, which shows potential for further clinical application.

## Introduction

Bone development displays certain age characteristics and crucial indicators that could directly reflect the level of maturity and biological age of an individual. Clinically, bone age assessment (BAA) is commonly used to diagnose and monitor growth disorders and endocrine abnormalities in children, including growth hormone deficiency (GHD), hypothyroidism, and precocious puberty, among others. Bone age (BA) can also be used in predicting adult height. It can even be a determining factor in whether a therapy is deemed necessary for patients with central precocious puberty (1, 2). With a whole host of practical applications, BAA has become a common clinical practice among pediatric endocrinologists.

Several methods are used globally in the evaluation of based BA on the left hand-wrist radiograph, including the Greulich-Pyle (GP) atlas and the Tanner-Whitehouse (TW) method (2, 3). Because these methods were largely developed based on a white population, an alternate method geared more toward the Asian population was developed by the Chinese Bone Development Survey Group. The China 05 (CH05) BA evaluation standard, which was formulated from 2003 to 2005 based on children from upper-middle backgrounds in developing cities around China, is now recommended by many experts as more suitable for evaluating Chinese children than the aforementioned methods (4–7).

As useful as it may be, the application of BAA comes with its drawbacks and limitations. Besides being time-consuming and challenging to master as a skill, the results of manual BAA highly depend on the clinician's level of experience. This can result in gaps between the results of BAA performed by junior- and senior-level clinicians, even when using the same set of radiographs. These problems will need to be addressed to ensure the accuracy of BAA, as it affects the diagnosis, monitoring, and treatment strategies of a number of diseases (7, 8).

In recent years, research on BAA has entered a new era with the arrival of artificial intelligence (AI). Several AI systems have already been developed to assess BA in North America and South Korea. These AI systems reportedly yielded both high accuracy and improved time-efficiency compared to manual assessment. However, most of the systems were based on the GP atlas or the TW3 method, which may not be applicable for the Chinese population (9). Although there has been some research involving AI BAA, to date, it has mainly comprised cross-sectional studies (9–11). To the best of our knowledge, no study has been done thus far to explore the performance of AI in longitudinal BA evaluations for endocrine disease, though it's sheer significant and common in clinical scenarios. This study, then, aimed to compare the accuracy and consistency of BAA among pediatric endocrinologists in the absence and presence of AI assistance during the course of treatment in children with GHD.

## Methods

### Participants

**Figure 1:**
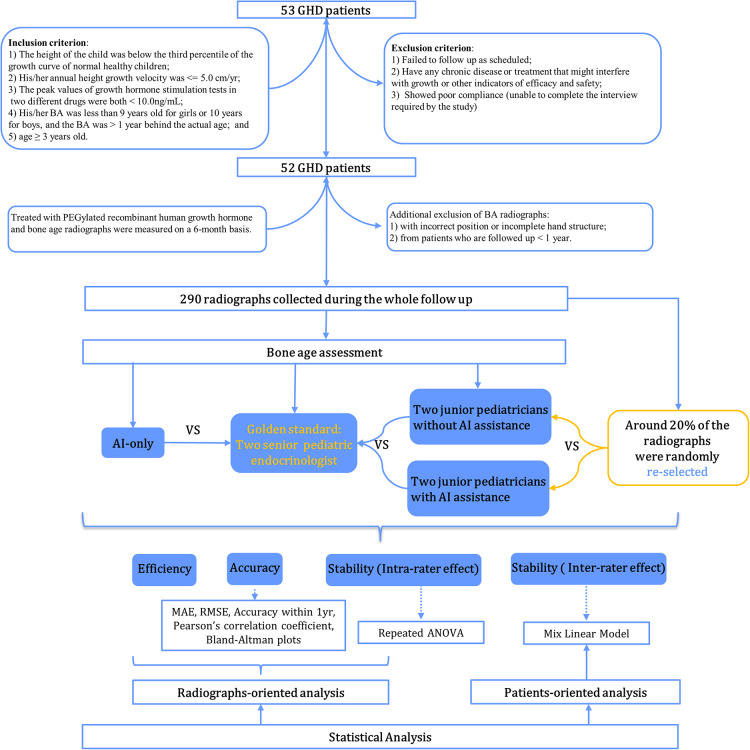
Flowchart of case selection and study scheme.

A total of 52 patients with GHD were prospectively enrolled between January 2016 and August 2017 at Sun Yat-sen Memorial Hospital. Study participants were treated with PEGylated recombinant human growth hormone for at least one year with a median follow-up period of 24 (IQR: 24–42) months. Inclusion criteria included the following: (1) the child's height at the first visit was below the third percentile of the growth curve for normal healthy children of the same age and sex; (2) the child's annual height velocity ≤5.0 cm/year; (3) the peak values of growth hormone (GH) stimulation tests of two different drugs <10.0 ng/ml, including complete GHD (cGHD) with a GH peak <5 ng/ml and partial GHD (pGHD)—GH peak in 5–10 ng/ml, and serum GH was measured using a solid-phase, 2-site chemiluminescent immunometric assay, Immulite 2000, and growth hormone assays were calibrated to NIBSC IS reference standard (98/574); (4) BA was below 9 years for girls or 10 years for boys and was more than one year behind chronological age; (5) prepubertal but ≥3 years old. Exclusion criteria included the following: (1) participants who did not follow up as scheduled or did not take the medication as directed (including injection dose and injection frequency); (2) those with any chronic disease or treatment that might interfere with growth or other indicators of efficacy and safety (such as the use of GnRH agonists, protein assimilation drugs, or long-term use of corticosteroids/traditional Chinese medicines, etc.); (3) subjects with poor compliance or who were unable to complete the interview as required by the study. Study participants' demographic characteristics, biochemical indicators, and BA radiographs were measured and monitored every 6 months. To compare the performance of BA readings longitudinally between pediatric endocrinologists and AI, additionally excluded were the following: (1) images with incorrect positioning (i.e., right hand) or with incomplete hand or wrist structures (such as no metacarpal bone, phalanx bone, carpal bone, or 3–4 cm of the distal shaft of the ulna and radius); (2) radiographs from patients who were followed up with for less than one year. Overall, 290 radiographs from 52 patients were collected and assessed. The flowchart of case selection is presented in Figure 1. The study was approved by the Ethics Committee of Sun Yat-sen Memorial Hospital (Ethics number: 2015–30), and all patients and their parents provided informed consent before participating.

### Inspection method

All radiographs were acquired using a YSID DR (Siemens, Germany) machine. The left palm of the subject was placed downward and gently pressed against the scanning platform, with all five fingers naturally separated. The angle between the thumb and index finger was about 30°, while the axis of the middle finger aligned with the forearm (the arm remained flat and could not be lifted), with a center line that was perpendicular to the head of the third metacarpal and a segment distance of 80 cm. A bandage was deployed if the subject was not able to position his or her hand properly without further assistance.

### AI system of BAA

**Figure 2:**
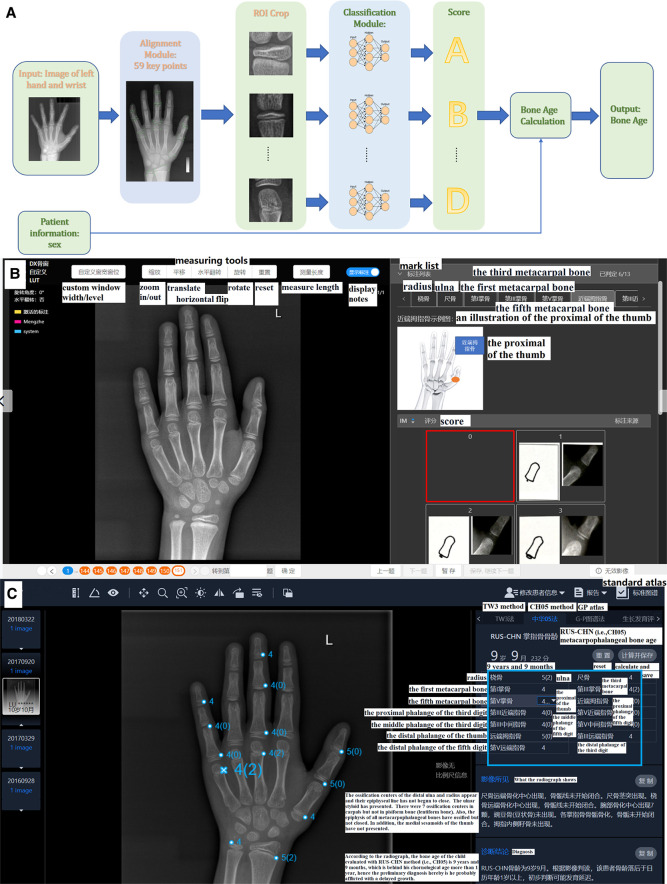
(**A**) Network structure of the region-based convolutional neural network. Two modules, including alignment and classification modules, adopted a deep residual network (ResNet) to evaluate bone age automatically. (**B**) The product window for doctors to evaluate bone age without AI assistance. Taking the proximal of the thumb as an example, the rater compares the patient's radiograph shown in the middle of the picture (by using the measuring tools in the upper middle such as zoom in/out) with the standard atlas (in the bottom right) and choses 0–8 points (marked as a red box) for it accordingly. In total, there are 13 ossification centers (i.e., the mark list as shown in the upper right including radius, ulna and so on) need to be scored and the bone age will be calculated automatically. (**C**) The product window for doctors to confirm and modify bone age with AI assistance. AI has scored the 13 ossification centers before raters’ evaluation (as presented in the blue box), and then raters begin to check each center according to atlas of CH05 which would show with a click of the button of standard atlas. When discrepancy appears, raters would re-compare with the standard atlas and decide to revise or remain the score accordingly. Finally, bone age is automatically calculated after a click of save button. Here the reader tends to score 4 points for the fifth metacarpal bone based on CH05 while it rated as 4(2) by AI, and a final 4(2) points was decided after a re-check with the standard atlas by the rater.

As illustrated in Figure 2A, the AI system (YITU Healthcare Technology Co., Ltd., China) used in this study comprised an alignment module and a subsequent classification module. Both modules were built on a deep residual network (ResNet), a deep convolutional neural network (CNN) with 50 layers and approximately 3.6 × 10^9^ floating point operations per second (FLOPS). The model was implemented using an open-source machine learning library (TensorFlow version 1.4.1; Google, Mountain View, CA, United States). The left hand and wrist images were automatically processed by the AI system, with targeted bones located, classified, and labeled (such as the radius, ulna, and metacarpals), maturity level evaluated, and BA calculated accordingly. It should be noted that the AI system supports BAA through the TW3 method (comprising 13 bones, i.e., the radius, ulna, and short finger bones), the RUS system (comprising 7 carpal bones), and the CH05 standard (6), since regions of interest may differ according to different medical scenarios. In the present study, AI-aided BAA was performed according to the CH05 standard, as it is more adapted to and thus preferred for assessing the skeletal growth patterns of Asian children (6). When AI-assisted BAA was conducted, the maturity level of each targeted bone was first estimated by AI, and then human raters could modify the outcome at will (Figure 2C). Likewise, manual BA evaluation was conducted on the same AI platform. The targeted bones were labeled by AI, but the maturity levels were assessed by raters alone without AI involvement (Figure 2B).

### Gold standard of BA

A total of 290 radiographs from the 52 study participants were randomly shuffled and independently evaluated by two senior pediatric endocrinologists (LLY and MZ, each with more than 15 years of experience in pediatric endocrinology) based on the CH05 standard, respectively. They were blinded to patient information, including age, gender, follow-up periods, and previous BA reports. Any inconsistent assessments of BA were re-evaluated, discussed, and confirmed with final consent from both raters. This rating outcome was referred to as the gold standard (GS).

### BAA with and without AI

Similar to the abovementioned procedure, two junior pediatric endocrinologists (ZLN and HLL, each with 5 years of experience in pediatric endocrinology) independently yet concurrently assessed the BA of the 290 radiographs. The slight distinction between their assessments and those of the senior pediatric endocrinologists involved the absence or inclusion of the AI-based BA evaluation system. Six months later, around 20% of the images were randomly selected to be re-evaluated by the junior pediatric endocrinologists both with and without AI assistance to measure intra-rater consistency over time. The rating process was not time-restricted, but the overall length of each evaluation was automatically recorded by the system. Here presents the detailed reading process of BA. Without AI: Based on standard of the CH05, readers evaluate the 13 ossification centers (i.e., radius, ulna, the metacarpals and the proximal, middle and distal phalanges of the first, third and fifth digits) and rate for them. The final bone age is calculated automatically with a weighted coefficient based on CH05. With AI: Before raters' evaluation, AI has rated the 13 ossification centers already, and then raters begin to read and rate on the basis of AI's score according to atlas of CH05. When discrepancy appears, raters would re-compare with the standard atlas of the CH05 and decide to revise or remain the score accordingly. Finally, the bone age is automatically calculated as well. As shown in Figure 2C, the reader rated 4 points for the fifth metacarpal bone based on the CH05 standard without AI assistance. However, AI rated it as 4(2). A final score of 4(2) points was determined by the reader after re-checking with the CH05 standard atlas. In this way, AI can help to improve the accuracy of BAA among less experienced clinicians.

### Statistical analysis

Clinical characteristics of the patients were described *via* mean and standard deviation (for normally distributed variables), interquartile range [median (Q25–Q75)] (for variables with non-normal distribution), or frequency and percentages (for categorical variables). The statistical analysis in this research was divided into two parts: (1) radiograph-oriented analysis to explore the accuracy, intra-rater effect, and efficiency of BAA; and (2) patient-oriented analysis to measure inter-rater variation during the course of GHD treatment. Several statistical variants were used to assess the BA divergence between the GS and the manual outcomes from the raters (or AI-only or AI-assisted) in radiograph-oriented analysis, including root mean square error (RMSE), mean absolute error (MAE), and accuracy within 1 year (%). Specifically, accuracy was defined as the percentage of the differences within 1 year. A paired *t*-test was used for MAE/RMSE, whereas McNemar's Chi-square test was used to check whether significant changes in those metrics were observed with or without AI assistance. Additionally, Pearson's correlation coefficient was calculated to measure their relativity, while Bland-Altman plots were generated to demonstrate the mean and 95% confidence interval of the differences. Fisher's *r* to *z* transformation was also performed, and a *Z*-test was used to compare the correlation coefficients. Furthermore, the intra-class correlation coefficient (ICC) based on two-way random ANOVA was used to assess inter-rater variation amongst the pediatricians with and without AI assistance as a measure of variability, while one-way repeated ANOVA was used to quantify the intra-rater effect between both instances of BA evaluation. As for patient-oriented analysis, a boxplot of BA evaluated by different raters during the course of treatment and a mixed linear model were used to illustrate inter-rater effect over time.

A two-tailed *P*-value of less than 0.05 was considered as statistically significant, and Bonferroni correction was applied if a statistical method was used multiple times. All analyses were conducted with R-3.6.2 (R Foundation for Statistical Computing, Vienna, Austria).

## Results

### Demographic characteristics of patients

A total of 52 children with GHD were included in this study. The mean chronological age of participants was 6.64 ± 2.49 years, while the mean BA as determined by GS was 5.85 ± 2.30 years at baseline (Table 1). Almost 6 in 10 (59.6%) subjects were girls whose mean height was 104.21 ± 13.39 cm, while the remainder were boys with a mean height of 112.49 ± 13.12 cm. Partial GHD accounted for 75% (*N* = 39) of all GHD cases.

**Table 1:** The demographic characteristics of the 52 GHD patients at the baseline.

Level	Overall	Boys	Girls	*P*
*n* (%)	52	21 (40.4)	31 (59.6)	
Age [mean (SD)]	6.64 (2.49)	7.44 (2.54)	6.10 (2.34)	0.039
Height [mean (SD)]	107.55 (13.78)	112.49 (13.12)	104.21 (13.39)	0.036
Weight [mean (SD)]	17.10 (5.08)	18.91 (5.38)	15.87 (4.56)	0.039
BMI [mean (SD)]	14.45 (1.13)	14.62 (1.18)	14.33 (1.10)	0.358
Ht SDS [mean (SD)]	−2.67 (0.85)	−2.60 (0.70)	−2.72 (0.96)	0.918
BA by GS [mean (SD)]	5.85 (2.30)	6.57 (2.68)	5.36 (1.90)	0.159
GHD type (%)				
pGHD	39 (75.0)	16 (76.2)	23 (74.2)	1.000
cGHD	13 (25.0)	5 (23.8)	8 (25.8)	

Ht SDS, height standard deviation scores; BA, bone age; GS, gold standard; cGHD, complete growth hormone deficiency; pGHD, partial growth hormone deficiency.

### Accuracy of BAA and AI effect on readers' performance

**Figure 3:**
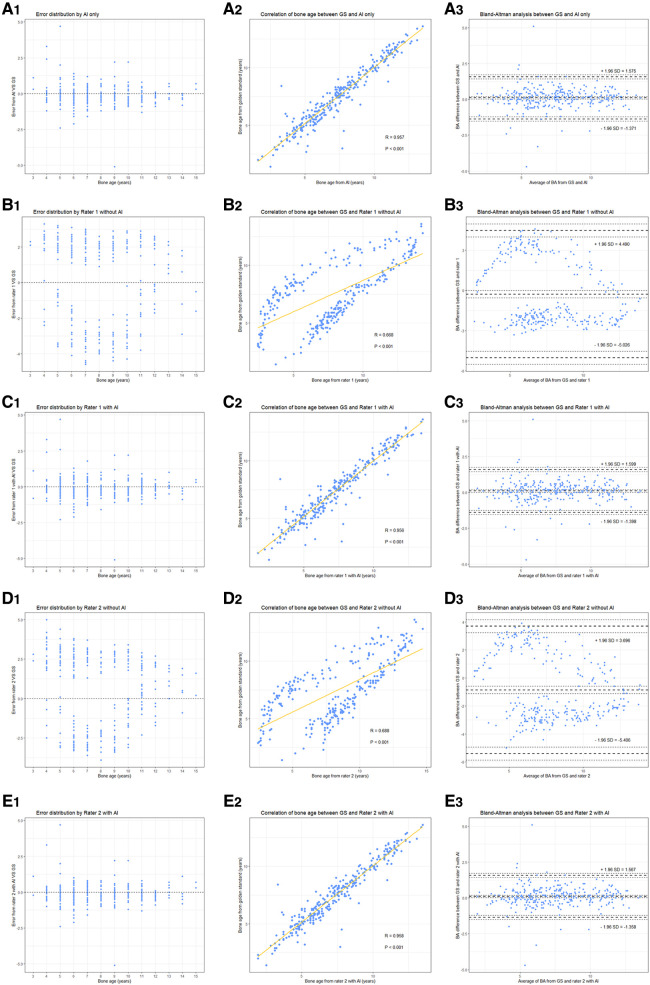
Performance of different raters in BA assessment. (left to right) Each column represents error distribution, correlation coefficient, and Bland-Altman analysis (95% LoA, limits of agreement), respectively. From top to bottom, i.e., (**A–E**) are the results performed by AI and the two junior raters (with or without AI assistance).

As presented in Table 2, the MAE and RMSE under AI assistance were 0.489 and 0.757 years, respectively, with an accuracy within one year of 91.03%, while the MAE and RMSE of both independent raters were more than two years off with an accuracy rate of around 10% (Rater 1: 8.36%; Rater 2: 13.45%). In addition, as shown in Figure 3, there was a significantly higher correlation (comparison *P* < 0.001) between AI-derived BA and the reference values (*r* = 0.957) than the BA as assessed by the two raters and GS (*r* = 0.668 and *r* = 0.688).

**Table 2:** Accuracy of BA assessment from different raters and effect of AI on raters’ performance.

	BA (mean ± SD, years)		MAE (years)		RMSE (years)		Accuracy within 1 year (%)	
	Without AI	With AI	Without AI	With AI	Without AI	With AI	Without AI	With AI
CA	7.866 ± 2.599	NA	NA	NA	NA	NA	NA	NA
GS	7.468 ± 2.591	NA	NA	NA	NA	NA	NA	NA
AI-only	NA	7.366 ± 2.548	NA	0.489	NA	0.757	NA	91.03
Rater 1	7.737 ± 3.205	7.368 ± 2.562	2.274	0.494	2.425	0.770	8.36	91.72
	*t* = 2.58	*P* = 0.010	*t* = −28.36	*P* < 0.001	*t* = −19.59	*P* < 0.001	*χ*^2^ = 229.33	*P* < 0.001
Rater 2	8.323 ± 3.144	7.364 ± 2.550	2.282	0.488	2.471	0.752	13.45	91.03
	*t* = 6.98	*P* < 0.001	*t* = −27.40	*P* < 0.001	*t* = −20.30	*P* < 0.001	*χ*^2^ = 215.35	*P* < 0.001

CA, chronological age; GS, gold standard; BA, bone age; NA, not applicable; MAE, mean absolute error; RMSE, root mean square error; *χ*^2^ here denotes McNemar's *χ*^2^ test.

With the mean BA values approaching to GS more clearly (for example, the BA by Rater 1 decreased from 7.737 ± 3.205 to 7.368 ± 2.562 when the GS is 7.468 ± 2.591), the performance of the two readers improved (all had *P* < 0.001) under the aide of AI, MAE and RMSE both decreased by more than 1.65 years (Rater 1: ΔMAE = 1.780, ΔRMSE = 1.655; Rater 2: ΔMAE = 1.794, ΔRMSE = 1.719) while accuracy increased to over 91% (Table 2). Notably, the application of AI resulted in higher accuracy (observed in Rater 1) and lower MAE and RMSE (observed in Rater 2) when combined with the pediatric endocrinologists’ interpretations as compared to AI alone. As illustrated in Figure 3, higher correlations (both had comparison *P* < 0.001) between the reference values and both readers (*r* = 0.956 and *r* = 0.958) could be observed upon the engagement of AI assistance. Bland–Altman plots revealed a decrease in the spread of ratings and decreased limits of agreement when paired with AI (Figure 3).

### Inter-rater and intra-rater variation with and without AI

ICCs were calculated to measure variations in inter-rater consistency both with and without AI assistance. The ICC between Rater 1 and Rater 2 without AI was 0.951 (95% CI, 0.830–0.978), which improved to 0.990 (95% CI, 0.987–0.992) with the assistance of AI. Overall, 62 radiographs were re-evaluated by the same raters both with and without AI. Repeated ANOVA showed significant variations (both with *P* < 0.001) between the initial and follow-up assessments of BA by the two junior pediatric endocrinologists. However, the significant differences disappeared (both have *P* > 0.0125) when the pediatric endocrinologists were assisted by AI (Table 3).

**Table 3:** Intra-rater effects in two times of bone age evaluation with and without AI.

Raters	Bone age (mean ± SD)		*F*-value	*P*-value
	First time	Second time		
Rater 1	8.39 ± 3.77	7.45 ± 3.58	13.066	0.001*
Rater 1 with AI	7.67 ± 3.28	7.45 ± 3.45	4.914	0.030
Rater 2	8.73 ± 3.68	7.76 ± 3.27	13.070	0.001*
Rater 2 with AI	7.63 ± 3.24	7.50 ± 3.30	1.760	0.190

Rater 1, Hou; Rater 2, Zhang.

* Denotes *P* < 0.0125 as Bonferroni correction was applied for four times of analysis using repeated ANOVA.

### Raters' effect in BA evaluation during the course of treatment

**Figure 4:**
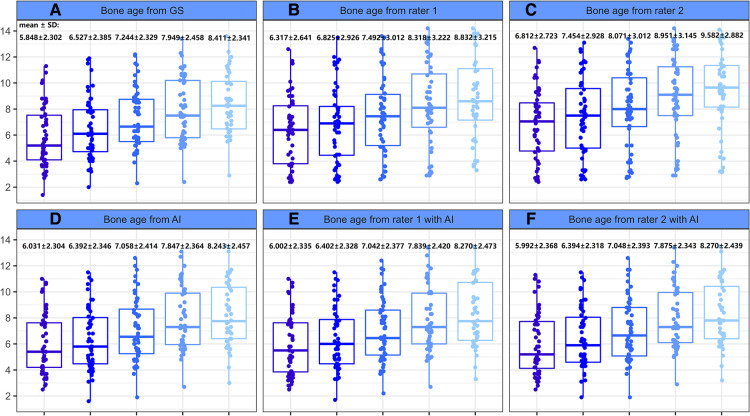
Boxplot of bone age as evaluated by different raters during the course of treatment. (**A**) Bone age from GS. (**B**) Bone age from rater 1. (**C**) Bone age from rater 2. (**D**) Bone age from AI. (**E**) Bone age from rater 1 with AI. (**F**) Bone age from rater 2 with AI.

The mean reference BAs for the 52 children at baseline, 6-, 12-, 18-, and 24-months were 5.848 ± 2.302, 6.527 ± 2.385, 7.244 ± 2.329, 7.949 ± 2.458, and 8.411 ± 2.341 years, respectively. Without AI assistance, BAs as assessed by Rater 1 and Rater 2 both had gaps greater than 2 years (Table 4) from the GS at all 5 time points, yet such differences decreased sharply upon application of AI. There was a significant rater effect (Rater 2 only) in the BA values without AI during the course of treatment, although no obvious interactive effect (rater*time) was observed whether or not AI was deployed (Table 4). A detailed distribution of BA assessment by different raters in the longitudinal follow-up is displayed in Figure 4.

**Table 4:** Raters effect in bone age evaluation in the course of treatment from analysis using mixed linear model.

Raters	△bone age (mean ± SD)					Rater's effect		Time effect		Raters*Time	
	Baseline	6 months	12 months	18 months	24 months	*b*	*P* value	*b*	*P* value	*b*	*P* value
AI VS GS	0.675 ± 1.103	0.558 ± 0.452	0.394 ± 0.337	0.463 ± 0.352	0.382 ± 0.287	0.122	0.330	0.677	0.000*	−0.069	0.076
Rater1 VS GS	2.327 ± 0.913	2.373 ± 0.787	2.308 ± 0.782	2.243 ± 0.863	2.239 ± 0.870	0.358	0.212	0.661	0.000*	−0.001	0.992
Rater1 with AI VS GS	0.758 ± 1.083	0.521 ± 0.523	0.398 ± 0.371	0.439 ± 0.334	0.386 ± 0.299	0.092	0.463	0.676	0.000*	−0.060	0.122
Rater2 VS GS	2.452 ± 0.985	2.242 ± 0.932	2.327 ± 0.983	2.280 ± 0.851	2.211 ± 1.047	0.838	0.002*	0.661	0.000*	0.046	0.590
Rater2 with AI VS GS	0.652 ± 1.070	0.571 ± 0.497	0.396 ± 0.360	0.455 ± 0.344	0.382 ± 0.290	0.077	0.539	0.675	0.000*	−0.053	0.170

GS, gold standard.

* Denotes *P* < 0.01 as Bonferroni correction was applied for five times of analysis using mixed linear model.

### Efficiency of rater-only and AI-assisted BAA

Overall, 290 radiographs were read by the 2 raters independently, which took 708 and 802 min to complete, respectively. When assisted by AI, their overall reading time decreased to 245 and 420 min, respectively, with the same number of images. Therefore, it could be said that AI helped to increase the pediatric endocrinologists’ work efficiency by almost two-fold.

## Discussion

In this study, the performance of an AI-based BA evaluation system was assessed during the course of endocrine treatment among children with GHD. Based on the results, AI was proven to significantly improve both the accuracy and consistency within and between raters in BAA. To date, this is the first known study to explore the potential application of AI in BA evaluation in more practical and clinically common scenarios.

This study's key finding implied that changes in BA values as assessed by the junior pediatric endocrinologists were significantly correlated with the raters during patient follow-up; even the BA rate showed inverse growth without AI. Delayed BA is a typical symptom of GHD, and BA catch-up is a common phenomenon of recombinant human GH therapy, which makes BAA useful in the evaluation of its therapeutic effect. However, the subtle BA changes during treatment can be difficult to detect by manual reading, especially in the absence of previous background information such as the diagnosis, results of previous ratings, or chronological age of the patient; as such, it is common to get a reverse increase in BAA (12). The determination of BA is commonly performed *via* visual comparison with the GP atlas or the TW3 method, so the outcome is prone to subjectivity. This occurs in part because when no perfect match exists in the reference material, readers must look for the reference image that exhibits the greatest similarity (10, 13), which is not conducive to the dynamic monitoring of BA and the therapeutic effects of treatment. The invention of AI assistance brings a possible, practical solution to this issue. As the results of this study suggest, BAA can be improved with the help of AI, which points to the benefits and significant clinical value of AI assistance in stable longitudinal BA monitoring. However, it should be noted that the results from this study were yielded by junior pediatric endocrinologists and excluded senior pediatricians and radiologists, so the results should be interpreted conservatively. Of note, clinical management of GHD treatment are based on height, height velocity and IGF levels, and BA recovery is only one of the referred factors. Hence, the process of BAA in follow-up, with or without AI, cannot replace the routine monitor of the indicators mentioned above.

This study demonstrated that the deployment of an AI assistance system decreased variations in inter-rater and intra-rater consistency. The results also built on recent data by Xi Wang et al., which demonstrated that an AI system based on CH05 BAA improved the performance of specialists with different levels of experience, thus increasing the ICC (14). The result also resonates with another study, which implied that an AI-assisted system could reduce variation in BAA by different raters, as well as the time required to read one radiograph (15). Improved consistency in BA evaluation would greatly benefit a variety of physicians and medical institutions in clinical practice, with the potential for increased precision in the diagnosis of endocrine disorders such as short stature (16), precocious puberty (17), and congenital adrenal hyperplasia (18).

Another goal of this study was to evaluate the efficiency and accuracy of BAA performed with the aid of AI. The results showed that not only could AI help to save time and increase the efficiency of BAA, which was a finding in common with previous studies (10, 14), but that the accuracy of both raters significantly improved after the assistance of an AI system (8.36% vs. 91.72%, 13.45% vs. 91.03%). The results of this study agreed with that of previous research where an automatic BAA system was used to rate the BA of Iranian children, which yielded an accuracy within a 1-year range of 95.32% for radiographs of female patients, and 96.51% for radiographs of male patients, respectively (19). Another study demonstrated an accuracy of 84.6% by applying an AI system based on the GP method among Chinese children with abnormal growth and development (11). It is worth mentioning that the accuracy of a rater with AI assistance was higher than that of AI alone (91.72% vs. 91.03%). It has been proven that readers can achieve better BA accuracy with the assistance of AI compared to either readers alone or AI alone (15, 20). The difference in accuracy may be related to the ability of readers to identify skeletal deformity and malposition from hand radiographs as they determine the BA. Specifically, since BAA is a subjective process and is susceptible to clinical experience, young pediatric endocrinologists may have difficulty in determining every score for each ossification center confidently, swaying in two grades sometimes. While with the aid of AI, whose training algorithm involves a quantitative process with each pixel of the image, such ambiguity may decrease and a better performance achieved correspondingly. It is of worth noting that BAA is more commonly done at 6–12 monthly intervals in clinical practice, and here in this study we chose the shortest one to monitor. Since the use of AI when added to the interpretation by junior pediatric endocrinologists improved the MAE and RMSE over this short time, if clinically used at a greater time interval such as yearly as may be done in clinical practice, this could result in an improve in accuracy of interpretation.

## Limitations

The data used in this study was limited to a single center within a single region, thus involving a relatively small data volume. The included data were mainly obtained from children between the ages of 3–13, with limited data taken from other age groups. This study only compared the values among junior pediatric endocrinologists under the absence and presence of AI assistance. In the future, the involvement of senior pediatricians and radiologists would be helpful in further elucidating the practicality and clinical value of AI assistance in BAA.

## Conclusion

In conclusion, the AI system in this study, which was constructed based on the CH05 BAA standard, was found to exhibit a high degree of accuracy with only slight deviation in the diagnosis and follow-up of GHD. With the increase of available sample data and further development of deep learning methods, the accuracy and efficiency of the automatic BAA can continue to be further expanded upon and improved. A future multi-center study will make BAA even more clinically adaptable.

## Data Availability

The datasets presented in this study can be found in online repositories. The names of the repository/repositories and accession number(s) can be found in the article/Supplementary Material.
